# CircRNAs and their regulatory roles in cancers

**DOI:** 10.1186/s10020-021-00359-3

**Published:** 2021-08-26

**Authors:** Mei Tao, Ming Zheng, Yanhua Xu, Shuo Ma, Weiwei Zhang, Shaoqing Ju

**Affiliations:** 1 Department of Laboratory Medicine, Affiliated Hospital of Nantong University, Xisi Road, No.20, Nantong, 226001 Jiangsu China; 2 Research Center of Clinical Medicine, Affiliated Hospital of Nantong University, Nantong, 226001 Jiangsu China; 3 Medical School of Nantong University, Nantong University, Nantong, 226001 Jiangsu China

**Keywords:** Circular RNA (circRNA), N-6 methylation (m6A), Biogenesis, Translation, Degradation, Cancers

## Abstract

Circular RNAs (circRNAs), a novel type of non-coding RNAs (ncRNAs), have a covalently closed circular structure resulting from pre-mRNA back splicing via spliceosome and ribozymes. They can be classified differently in accordance with different criteria. As circRNAs are abundant, conserved, and stable, they can be used as diagnostic markers in various diseases and targets to develop new therapies. There are various functions of circRNAs, including sponge for miR/proteins, role of scaffolds, templates for translation, and regulators of mRNA translation and stability. Without m7G cap and poly-A tail, circRNAs can still be degraded in several ways, including RNase L, Ago-dependent, and Ago-independent degradation. Increasing evidence indicates that circRNAs can be modified by N-6 methylation (m6A) in many aspects such as biogenesis, nuclear export, translation, and degradation. In addition, they have been proved to play a regulatory role in the progression of various cancers. Recently, methods of detecting circRNAs with high sensitivity and specificity have also been reported. This review presents a detailed overview of circRNAs regarding biogenesis, biomarker, functions, degradation, and dynamic modification as well as their regulatory roles in various cancers. It’s particularly summarized in detail in the biogenesis of circRNAs, regulation of circRNAs by m6A modification and mechanisms by which circRNAs affect tumor progression respectively. Moreover, existing circRNA detection methods and their characteristics are also mentioned.

**Supplementary Information:**

The online version contains supplementary material available at 10.1186/s10020-021-00359-3.

## Introduction

It was a long time after circRNAs were discovered (Nigro et al. 1991; Hsu and Coca-Prados 1979), but researchers paid little attention because they considered them spliced in error (Cocquerelle et al. 1993; Hentze and Preiss 2013). However, with the development of high-throughput sequencing, a growing number of circRNAs are discovered in many species (Jeck et al. 2013; Jeck and Sharpless 2014; Chen et al. 2018a; Memczak et al. 2013). In 2013, Memczak et al. confirmed that circRNAs of the nervous system form a class of post-transcriptional regulators that compete with other RNAs in binding to miRNAs and RBPs, which ultimately affects the development of the nervous system (Memczak et al. 2013). Attention has been increasingly paid to the study of circRNAs ever since, and the characteristics and significance of circRNAs have come to light. CircRNAs are abundant (Westholm et al. 2014; Rybak-Wolf et al. 2015) and most located in the cytoplasm and very few in the nucleus (Jeck et al. 2013; Zhang et al. 2013). Besides, they have better stability than linear mRNAs as the former can resist the degradation of exonuclease and have a longer half-life period (Cocquerelle et al. 1993). High abundance and good stability make it possible to use circRNAs as biomarkers in various diseases. CircRNAs have numerous functions, and the review will discuss their functions as sponge, templates for translation, role of scaffold and mRNA regulators in detail.

Posttranscriptional modification is important to life. m6A is an RNA modification found in many species up to now, which is significant for RNA regulation (Dominissini et al. 2012; Schwartz et al. 2013). The majority of m6A sites are observed in the vicinity of the stop codon and the 3′UTR (Dominissini et al. 2012; Zhou et al. 2015; Meyer et al. 2012). The regulation of m6A is a dynamic system, which is composed of methyltransferases (WTAP, METTL3 and METTL14) that act as writers, readers (YTHDF domain-containing proteins like YTHDF1/2/3 and IGF2BPs) that can recognize m6A, and erasers (FTO and ALKBH5) that eliminate the function of m6A (Zhao et al. 2020a). To date, many researchers have demonstrated that m6A regulates mRNAs in a lot of ways such as promoting translation and degradation of mRNAs (Zhou et al. 2015; Shi et al. 2017; Lee et al. 2020), which may contribute to various diseases in humans. There are few studies on the post-transcriptional modification of circRNAs, but m6A modification of circRNAs is becoming a hot research topic. This paper summarizes various effects of m6A modification on circRNAs, including biogenesis, degradation, nuclear export and translation.

Additionally, the regulatory effect of circRNAs on tumor progression has been confirmed in many tumors. This paper also summarizes the regulatory mechanisms of circRNAs for tumors from different systems in detail, providing a rich theoretical basis for clinical research.

Briefly, the review summarizes biogenesis, biomarker, functions, degradation, m6A modification, regulation in cancers, and detection methods of circRNAs in a detailed way.

## Biogenesis of circRNAs

**Fig. 1:**
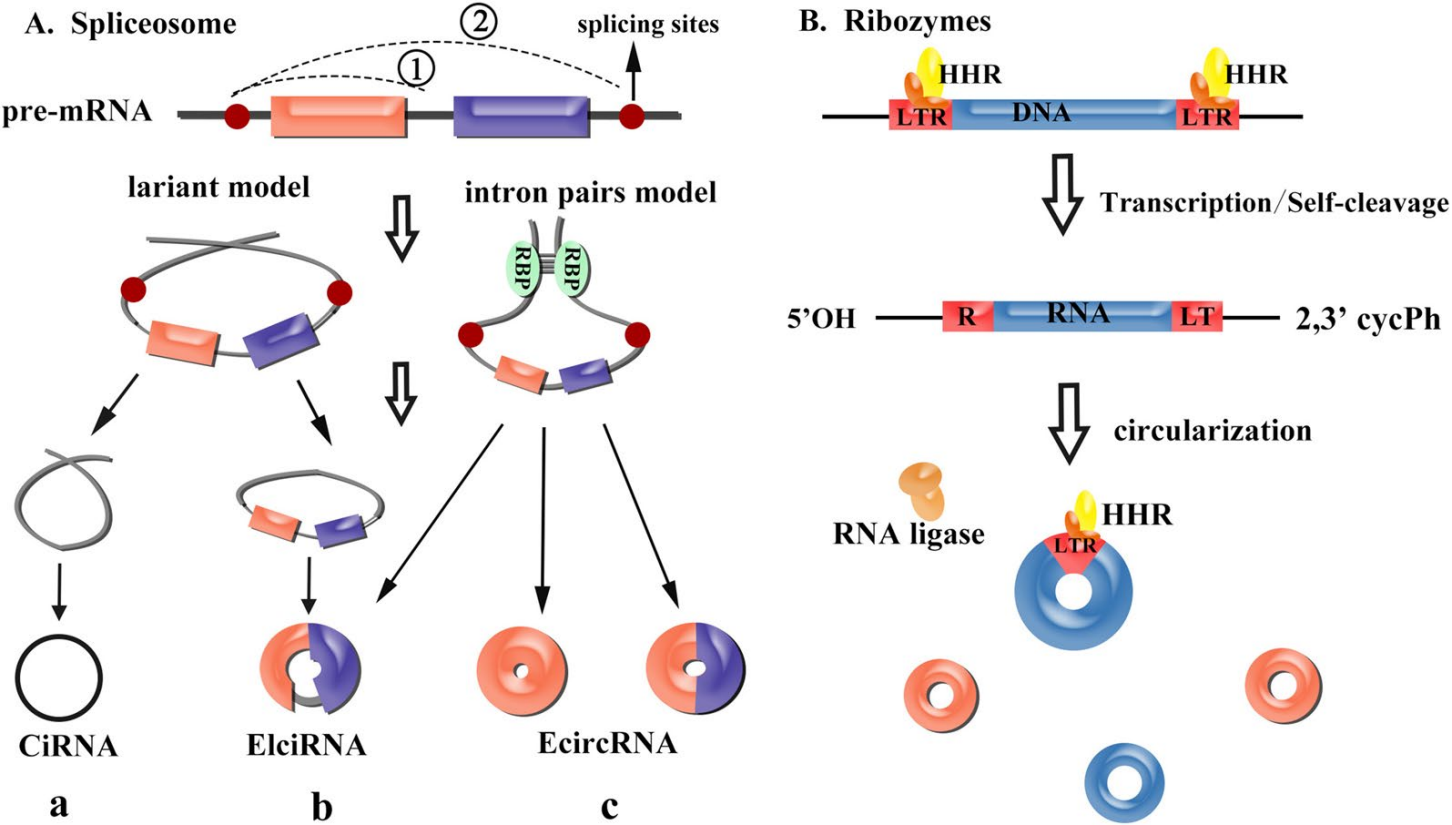
Biogenesis of circRNAs. **A** Lariat-Driven Circularization model and Intron-Driven Circularization model. The former produces a lariat because of exon skipping. The lariat makes the splicing sites of circularized exons closer than before. Then, the lariat is cut again by the exons; in this way, a circRNA and a free lariat composed of introns are produced. The latter reveals that repetitive and reverse complementary sequences in flanking introns form a stem-loop structure, which brings the splicing sites into spatial proximity. Based on composition, circRNAs are divided into 4 types including **(a)** CiRNAs formed by introns merely, **(b)** EIciRNAs with both exons and introns inside the circular structure and **(c)** EcircRNAs with only exons to circularize. In addition, assume that one splicing site is stationary then alternative back splicing can produce two different circRNAs, as shown in dotted lines. Products of ① corresponds to the left one in **(c)** and ② corresponds to the right one. **B** Hammerhead ribozymes (HHRs) are embedded in direct long terminal repeats (LTRs) of DNA. After transcribed into RNA, HHR catalyzes the formation of 5’OH and 2,3′ cycPh. Then RNA ligase works to form the circular structure of linear RNA

CircRNAs are formed in two different ways, including spliceosome and ribozymes, as shown in Fig. 1.

For the first one, circularization of exons results from back splicing of pre-mRNA. There are two classical models of circularization: the Intron-Driven Circularization and the Lariat-Driven Circularization (Jeck et al. 2013). The former reveals that repetitive and reverse complementary sequences such as Alu elements in flanking introns tend to form a stem-loop structure, bringing the splicing sites into spatial proximity to facilitate the formation of circular structures (Ivanov et al. 2015; Dubin et al. 1995; Zhang et al. 2014; Liang and Wilusz 2014). Evidence shows that compared to the exons with shorter flanking introns, the exons with longer flanking introns are easier to circularize as they can harbor more repetitive and reverse complementary sequences (Vo et al. 2019). The latter produces a lariat because of exon skipping. After the lariat makes the splicing sites of circularized exons closer than before, it is cut again by the exons; finally, a circRNA and a free lariat composed of introns are produced (Jeck et al. 2013).

Besides spliceosome, it’s reported that hammerhead ribozymes (HHRs) also promote the circularization of circRNAs (Peña 2018). HHRs are embedded in direct long terminal repeats (LTRs) of ~ 350 bp. When a genomic retrozyme with at least 2 HHRs is transcribed to an RNA, the RNA forms 5-OH and 2,3-cyclic phosphate ends by self-processing through HHRs. Subsequently, an RNA ligase works to form the circular structure of linear RNA (Peña 2018). Notably, the ability of ribozymes to produce circRNAs has been used to amplify circRNAs in vitro (Litke and Jaffrey 2019). The difference between spliceosome and ribozymes is that complementary base pairing and spatial proximity between splicing sites are necessary for the former, but not for the latter.

### Regulation of circRNA biogenesis

Studies show that the circularization of exons can be regulated. For positive regulation, RNA binding proteins (RBPs) promote circularization by reducing spatial proximity as most of them bind to their binding sites in flanking introns (Ashwal-Fluss et al. 2014; Conn et al. 2015; Dai et al. 2018). Besides, the upstream promoter of the circRNA is activated under some circumstances, which also contributes to the formation of the circRNA (Zhao et al. 2020b; Wang et al. 2018; Sun et al. 2020a). However, it’s reported that circularization can be promoted by most RBPs except DHX9, which inhibits the production of circDLC1 as it can bind to the flanking reverse complementary sequence of circDLC1 and inhibit the pairing of these sequences (Liu et al. 2021). Besides, ADAR1, one of the RNA editing enzymes, was found to restrict the circularization of exons due to A-to-I editing events that destroy interaction in the base pair of introns (Ivanov et al. 2015; Shi et al. xxxx). Meanwhile, the production of circular RNA can also be brought down by G-U wobble base pairs and poly(A) stretches in repeat sequences, etc. (Liang and Wilusz 2014).

### Alternative splicing and alternative back splicing

Pre-mRNA splicing is found to happen in eukaryotes (Kim et al. 2007). When it happens, non-coding introns are removed, and the protein-coding elements are assembled to form a mature messenger RNA (mRNA) (Sharp 1994). Theoretically, a limited number of genes in eukaryotes can’t provide sufficient proteins for life activities. On the contrary, researchers discovered alternative pre-mRNA splicing, a mode that can be regulated and introduces protein diversity to eukaryotes (Black 2000; Graveley 2001).

There is another pre-mRNA splicing named alternative back splicing (Zhang et al. 2016; Gao et al. 2016; Pagliarini et al. 2020). Researchers revealed that a single gene locus could produce multiple circular RNA transcripts called alternative circularization, which results from the alternative formation of inverted repeated Alu pairs (IRAlus) and the competition between them. This may be the reason for the structural diversity of circRNAs. Interestingly, alternative splicing in circRNAs prefers nucleus localization to cytoplasmic localization and exhibits tissue- and developmental stage-specific expression patterns (Gao et al. 2016). Based on the alternative back splicing site selection, alternative back-splicing is categorized as alternative 5′ back-splicing and alternative 3′ back-splicing. Moreover, there have been four canonical types of alternative splicing found in multiple-exon circRNAs (Zhang et al. 2016).

It is reported that circRNA-forming back splicing is performed by spliceosome that conducts canonical splicing, resulting in competition between the back and canonical splicing (Ashwal-Fluss et al. 2014; Starke et al. 2015). Particularly, back splicing may take more time than pre-mRNA linear splicing due to the base pair in introns or recruitment of the spliceosome (Liang and Wilusz 2014).

### Co-transcriptionally or post-transcriptionally

When it comes to circularization occurring co- or post-transcriptionally, researchers hold contradictory opinions with each other (Liang and Wilusz 2014; Ashwal-Fluss et al. 2014; Kramer et al. 2015). Khodor et al*.* validated that most S2 cell introns and fly head introns in Drosophila can be efficiently spliced co-transcriptionally by sequencing nascent RNA transcripts. Besides, a greater co-transcriptional splicing was detected in a slower Pol II elongation rate (Khodor et al. 2011). The two findings both provide support for co-transcription. However, Liang et al. had the opposite discovery that the expression plasmid can’t produce circRNAs without pre-mRNA 3′ end formation (Liang and Wilusz 2014). Furthermore, circRNAs could be produced at higher level when the linear transcript had a stable 3′ end. They concluded that circularization might occur post-transcriptionally (Liang and Wilusz 2014). Later, a reasonable explanation concerning this contradiction was proposed (Kramer et al. 2015). Marianne et al*.* elucidated that the key difference between their genes in the above studies was that they had different intronic repeats. Specifically, they discovered that long repeats (300 nt or longer) in the flanking introns were likely to promote co-transcriptional back splicing (Kramer et al. 2015).

## Classifications of circRNAs

Up to now, there have been several classifications of circRNAs based on different standards. Firstly, according to the components of circRNAs, they are classified as EcircRNAs only made up of exons (Jeck et al. 2013), CiRNAs formed by introns merely (Zhang et al. 2013), and EIciRNAs composed of both exons and introns (Li et al. 2015). Secondly, most circRNAs are derived from single gene, but evidence shows that they can also originate from different genes (Vo et al. 2019; Guarnerio et al. 2016). F-circRNAs are resulted from gene fusions, in which cyclized exons are from both genes (Guarnerio et al. 2016). Rt-circRNA (read-through circRNA) is a newly found circRNA that is formed by exons from two adjacent genes on the same strand (Vo et al. 2019**).** Thirdly, they can also be divided into nuclear circRNAs and cytoplasmic ones based on their cellular localization. It is reported that the majority of circRNAs are located in the cytoplasm (Jeck et al. 2013; Memczak et al. 2013). Different distribution in cells gives circRNAs different roles in life activities, which means that sometimes circRNAs need to be transferred to the nuclear from the cytoplasm or the other way round. However, further investigation is needed to reveal the mechanisms.

## Functions of circRNAs

**Fig. 2:**
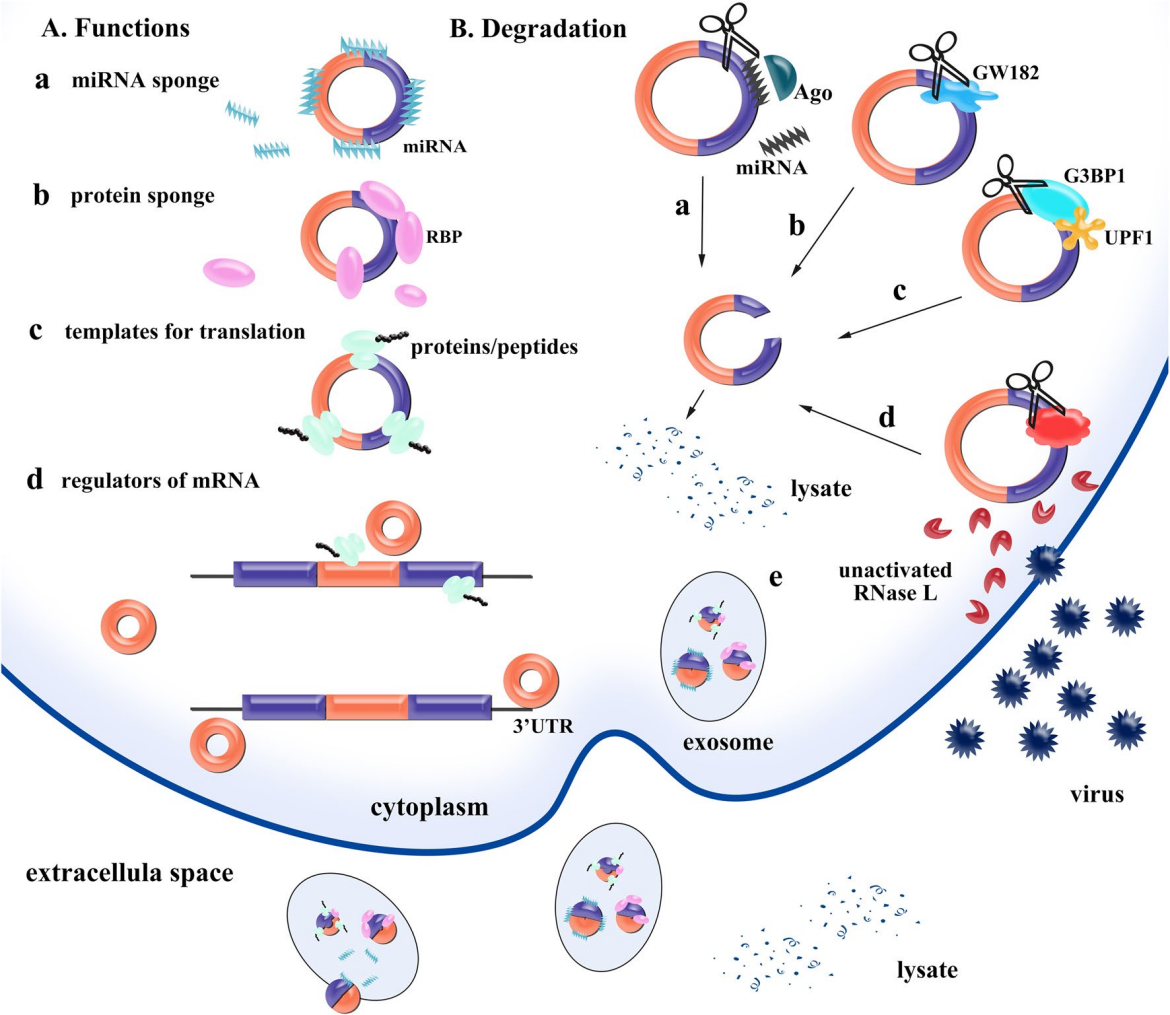
Functions and degradation of circRNAs. **A** Functions of circRNAs **(a)**. CircRNAs play a regulatory role by binding to miRNAs **(b)**. CircRNAs bind to proteins to act as a protein sponge or a protein scaffold **(c)**. CircRNAs can be translated into proteins or peptides in cap-independent way, during which IRES or other initial elements are necessary **(d)**. CircRNAs can be regulators of mRNA, including regulating translation of mRNA (up) and stability of mRNA (down). **B** Degradation of circRNAs **(a)**. miR-671 is located in nucleus and directs the Ago2-slicer-dependent cleavage of circCDR1 **(b)**. A middle region (Mid) in GW182 functions as a molecular scaffold to recruit decay factors, resulting in degradation of circRNAs **(c)**. Highly structured circRNAs can be degraded by UPF1 and G3BP1. Both proteins are indispensable in this progress (**d**). RNase L can be activated once cells are invaded by virus. Subsequently, activated RNase L mediates the global degradation of circRNAs, which is essential to activate PKR in the early stage of the innate immune response **(e)**. Cell excretion of circRNAs into the extracellular space through extracellular vesicles (EVs) may be a mechanism for cell clearance of circRNAs

Functions of circRNAs are summarized in Fig. 2A.

**Fig. 3:**
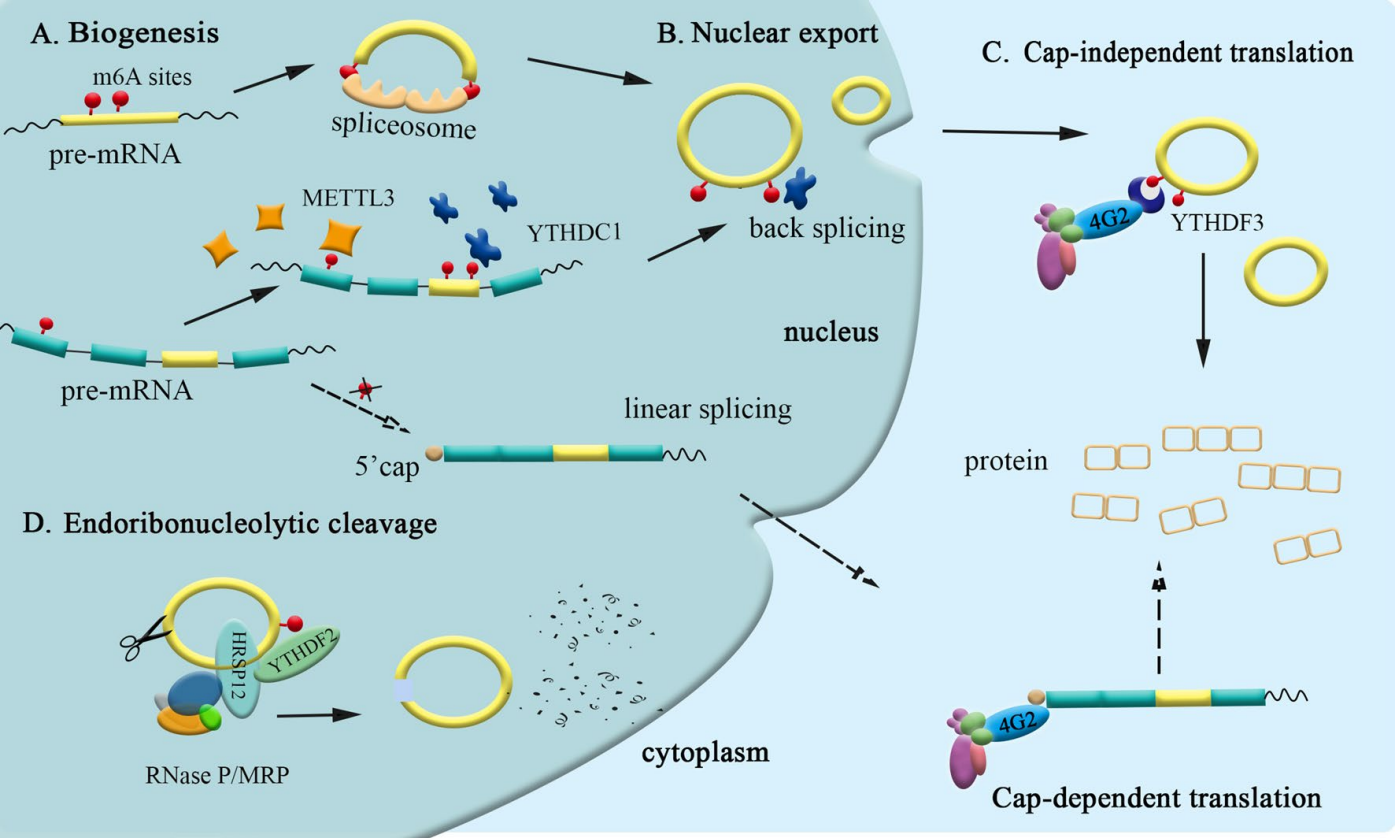
Roles of m6A modification in circRNAs. **A** m6A controls biogenesis of circRNAs. Pre-mRNAs with m6A sites recruits spliceosome or enzymes associated with methylation to promote back splicing of pre-mRNAs, while pre-mRNAs without m6A sites go through linear splicing to become linear mRNAs. **B** YTHDC1 binds to m6A sites in circRNAs to help circRNA nuclear export. **C** Different from translation initiation complex (IC) that binds to 5′ cap to initiate translation of mRNAs, IC binds directly to m6A sites that function as an IRES to initiate translation of circRNAs in a cap-independent way. **D** CircRNAs can be degraded through endoribonucleolytic cleavage way, where HRSP12 bridges between RNase/MRP and YTHDF2

### Sponge

Sponge, also known as competitive endogenous (CE) function, is the most mature function that has been studied of circRNAs by far. It’s Memczak that first brought up the description of the circRNA sponge. In 2013, Memczak et al. found 63 binding sites between the cerebellar degeneration-related protein 1 (circCDR1) and miR-7. CircCDR1 impaired brain development by sponging miR-7 via these binding sites (Memczak et al. 2013). Based on the finding hereinabove, the function of circRNAs as post-transcriptional regulators was gradually recognized and accepted, and sponge is a common way of regulation. CircPRMT5 is upregulated in serum and urine exosomes from urothelial carcinoma of bladder (UCB) patients. Its function is to modulate the SNAIL1 / E-cadherin pathway in order to promote epithelial-mesenchymal transition (EMT) in UCB cells via competitive combination with endogenous miR-30c (Chen et al. 2018b). Similarly, it was reported that circSnx5 sponged miR-544 to mitigate miRNA-mediated inhibition of cytokine signaling 1(SOCS1), leading to inhibition of dendritic cells (DC) maturation and accelerated the formation of Treg at the same time (Chen et al. 2020c).

Along with sponging miRNAs, circRNAs feature post-transcriptional regulation by sponge proteins. For example, circSnx5 can sponge not only miR-544 but also PU.1 to suppress its nuclear translocation, and finally inhibit the transcription of PU.1 and MHC class II expression in downstream DCs (Chen et al. 2020c). Similarly, circSORE binds to YBX1 in the cytoplasm to prevent YBX1 translocation to the nucleus for interaction with PRP19, avoiding subsequent degradation of YBX1 by PRP19 (Xu et al. 2020a). As a nuclear circRNA, circHuR interacts with CCHC-type zinc finger nucleic acid-binding protein (CNBP) and inhibits its binding to the HuR promoter, which ultimately down-regulates HuR and inhibits gastric cancer (GC) progression (Yang et al. 2019). Additionally, circFOXP1 regulates the Warburg effect by interacting with PTBP1 to up-regulate the expression of PKR and exert its pro-tumor effect, eventually protecting PKLR from mRNA degradation (Wang et al. 2019a). Likewise, circCDKN2B-AS1 promotes the malignant phenotype of cervical cancer by sponging the IMP3 protein to stabilize HK2 mRNA, thereby accelerating aerobic glycolysis (Zhang et al. 2020a).

In conclusion, the sponge function of circRNAs is universal and significant, and their importance as post-transcriptional regulators is worth attention.

### Scaffold

A growing number of studies indicate that circRNAs act as scaffolds in the formation of functional complexes. Evidence shows that circSKA3 expression speeds up tumor progression as well as the formation of invadopodium (Du et al. 2020). Further studies indicate that TKS5 and Integrin β1 are binding partners of circSKA3, which is a key molecule in the binding of ITGB1 and TKS5 to form invadopodium. It functions as a scaffold that combines TKS5 and Integrin β1 to induce the formation of invadopodium (Du et al. 2020). Normally, cyclin-dependent kinase 2 (CDK2), the cell cycle proteins, interacts with cyclins A and E to facilitate cell cycle entry, while p21 inhibits these interactions and ends up blocking cell cycle progression. In the meantime, circFoxo3 functions as a scaffold to bind to CDK2 and p21 and form a ternary complex that inhibits CDK2 function and prevents cell cycle progression (Du et al. 2016a). Similar to circFoxo3, other circRNAs also play the role of the scaffold. For example, circLRIG3 physically interacts with EZH2 and STAT3 and acts as a scaffold to increase EZH2-induced STAT3 methylation and subsequent phosphorylation. In turn, activated STAT3 directly binds to the circLRIG3 promoter region, which enhances the circLRIG3 transcription, and at last, forms a positive regulatory circuit that improves hepatocellular carcinoma (HCC) processes (Sun et al. 2020a). Another example is circNDUFB2 that binds to the IGF2BPs KH domain and acts as a stent to enhance the interaction between TRIM25 and IGF2BPS, eventually expediting TRIM25-mediated IGF2BPS ubiquitination and degradation (Li et al. 2021a).

Another important function of circRNAs is that the scaffold is indispensable. Suppose that elements A and B can be combined more closely and are more powerful after binding together to scaffold, but competitive against each other functionally, the scaffold can combine element C that will bind to one of them so that the other can perform its function.

### Templates for translation

CircRNA, a kind of non-coding RNAs, had been considered untranslatable for a long time. However, more and more circRNAs were revealed to have a function of peptide or protein coding in the past few years. There are two initiation mechanisms of protein synthesis in RNA: cap-dependent and cap-independent translation initiation (Filbin and Kieft 2009; Aitken and Lorsch 2012; Kwan and Thompson 2019). For the former, eukaryotic initiation factor (eIF) proteins and the small (40S) subunit of the ribosome bind to a modified nucleotide cap on the mRNAs 5′end. Subsequently, the complex begins to scan to find the start codon. Finally the large (60S) ribosomal subunit is recruited to form a ribosome to translate (Kwan and Thompson 2019). Therefore, 5′ cap is necessary in cap-dependent translation initiation. Due to the lack of 5′ cap, circRNAs can't be translated this way.

On the other hand, the second mechanism, known as cap-independent translation initiation, is independent of cap structure. During this process, translation is driven by some particular elements. Specific RNA sequences called internal ribosome entry sites (IRESs) have been confirmed to initiate translation in many cases (Yang et al. 2018; Xia et al. 2019). Different from cap-dependent translation initiation, the 40S can directly bind to the IRES element to initiate the translation of circRNAs (Chen and Sarnow 1995).

It’s worth noting that there is another cap-independent translation called rolling circle translation, which has been demonstrated by using synthetic circRNAs (Abe et al. 2015). It has been found in eukaryotic cell lines and a prokaryote system (Abe et al. 2013, 2015). Compared to linear mRNAs, circRNAs can be more efficiently translated through a rolling circle amplification (RCA) mechanism in a cell-free Escherichia coli translation system. With an infinite open reading frame (ORF) and no stop codons, circRNAs can be translated more efficiently than linear counterparts even without initial driving elements, indicating that another function of circRNAs is template for rolling circle translation.

Translation of circRNAs itself can be regulated. Poly-A tails at the 3′ end of mRNA were reported to enhance the translation of linear RNA (Abe et al. 2013). However, in Wand’s research, the translation of circRNAs was efficiently inhibited when poly(A)/poly(T) fragment was inserted after the stop codon of GFP in circRNAs, indicating that endogenous circRNAs with these features might incur translation inhibition (Wang and Wang 2015). Based on such a finding, it can be concluded that the translation of circRNAs can be regulated.

### Regulators of mRNA

#### Regulate translation of mRNA

CircRNAs can be not only translated into proteins, but also used as regulator of mRNA translation. When circMALAT1 binds to the ribosome via IRES and to the coding sequence of PAX5 mRNA via the 11-base complementary sequence, a specific triple complex (ribosome-circRNA-mRNA) is formed. The complex directly blocks the translation of PAX5 mRNA and affects PAX5-related cellular functions by embedding circMALAT1 between the PAX5 coding sequence and the ribosome like a brake (Chen et al. 2020d). In addition to circMALAT1, circYAP is another circRNA reported to regulate mRNA translation (Wu et al. 2019a). In the translation-initiation complex, eIF4G and PABP, which are responsible for the initiation of YAP translation, trigger mRNA cyclization and support for translation when they bind to each other. Meanwhile, circYAP can specifically recognize and bind to YAP mRNA, and eliminates the interaction between eIF4G and PABP after simultaneously binding to them each, resulting in inhibiting the initiation of YAP translation (Wu et al. 2019a). Likewise, circMYBL2 promotes FLT3 kinase translation by strengthening the binding of PTBP1 to FLT3 mRNA, which regulates FLT3 kinase level and activation of FLT3-ITD-dependent tumor signaling pathways (Sun et al. 2019).

#### Regulate stability of mRNA

Evidence shows that circRNAs can regulate the stability of mRNA at the same time. When circXPO1 binds to IGF2BP1 and enhances the stability of CTNNB1 mRNA, CTNNB1 inhibition is increased and lung adenocarcinoma progression is promoted (Huang et al. 2020a). Similarly, circARHGAP12 promotes the progression of nasopharyngeal carcinoma (NPC) by binding to 3′ UTR of EZR mRNA, which enhances the stability of EZR mRNA (Fan et al. 2021).

## CircRNAs as biomarkers

Thousands of reproducible circRNAs were identified by whole-transcriptomic analysis of human peripheral blood, and the expression of these circRNAs was higher than that of linear transcripts, laying the foundation for subsequent studies about biomarker potential of circRNAs in tumors (Memczak et al. 2015). CircRNAs have potential to be biomarkers because of following characteristics. Firstly, circRNAs have good stability and long half-life because of their covalently closed circular structure (Cocquerelle et al. 1993). Compared to linear transcripts, circRNAs can resist RNase R degradation (Jeck et al. 2013; Memczak et al. 2013; Suzuki et al. 2006) and have a longer half-life of more than 48 h (Cocquerelle et al. 1993; Jeck et al. 2013; Enuka et al. 2016). Secondly, they are genetically conserved and expressed in high abundance (Jeck et al. 2013; Memczak et al. 2013; Rybak-Wolf et al. 2015; Salzman et al. 2012), even 10 times than that of the corresponding linear mRNAs. In addition, circRNAs are widespread in blood (Memczak et al. 2015; Koh et al. 2014; Vea et al. 2018), saliva (Bahn et al. 2015; Zhao et al. 2018), urine (Chen et al. 2018b; Kölling et al. 2019), and gastric juice (Shao et al. 2017), which may provide more options for circRNAs to become ideal biomarkers. Thirdly, the expression pattern of circRNAs is specific, including cell-specific, tissue-specific and development-specific (Memczak et al. 2013). Different mature tissues express a certain number of unique circRNAs (Xu et al. 2017), and this process is independent of the expression level of corresponding host genes (Zhou et al. 2018). Besides, studies have shown that circRNAs accumulate gradually in the process of brain aging in an age-dependent manner (Zhou et al. 2018; Szabo et al. 2015), suggesting that circRNAs may be potential biomarkers of aging. Taken together, circRNAs are expected to be utilized as ideal biomarkers and therapeutic targets in clinic.

It has been demonstrated in a variety diseases that plasma circRNAs can be potential biomarkers. Hsa_circ_0005402 and hsa_circ_0035560 can serve as biomarkers for multiple sclerosis (Iparraguirre et al. 2017) while the combination of hsa_circ_0001879 and hsa_circ_0004104 for coronary artery disease (Wang et al. 2019b). Moreover, plasma circRNAs can serve as biomarkers for a number of tumors. For example, hsa_circ_002059, hsa_circ_0000520, and hsa_circ_0000190 are considered non-invasive diagnostic markers for gastric cancer (GC) (Li et al. 2015b; Sun et al. 2018; Chen et al. 2017). CircFARSA and the combination of hsa_circ_0005962 and hsa_circ_0086414 are considered as novel diagnostic markers for lung cancer (Hang et al. 2018; Liu et al. 2019a). Notably, hsa_circ_0013958 is more prominent in the early diagnosis and screening of lung cancer (Zhu et al. 2017). In hepatocellular carcinoma (HCC), the expression level of hsa_circ_0001445 in plasma can be used for differential diagnosis of HCC (Zhang et al. 2018a), while that of circSMARCA5 can predict and monitor the progression of HCC (Li et al. 2019b). In addition, dynamic monitoring of plasma hsa_circ_0005397 helps predict postoperative recurrence and metastasis of HCC (Liu et al. 2021). Others like circLDLRAD3, hsa_circ_0066755, hsa_circ_0001649, and hsa_circ_0001785 are respectively used for the diagnosis of pancreatic, nasopharyngeal, colorectal, and breast cancer (Yin et al. 2018; Yang et al. 2017a; Wang et al. 2020; Ji et al. 2018).

In addition to biomarkers, circRNAs can also be targets for tumor therapy. Hsa_circ_001659 is a serum biomarker for the early diagnosis and prognosis of colorectal cancer (CRC) (He et al. 2021a). Further studies showed that hsa_circ_001659 recruited RBBP5 to the vimentin promoter and increased the level of H3K4 trimethylation in the vimentin promoter region, thus activating the transcription of vimentin. Therefore, hsa_circ_001659 is a potential biomarker as well as a therapeutic target for CRC (He et al. 2021a). In non-small cell lung cancer (NSCLC), circPTEN (hsa_circ_0094342) has low expression in tissues and serum, and is associated with malignant clinical characteristics and poor prognosis (Wang et al. 2021). When it increases the expression of its host gene PTEN by acting as a sponge for miR-155 and miR-330-3p, the oncogenic PI3K/AKT signaling pathway is inactivated, which finally inhibits cell proliferation and tumor growth. Therefore, hsa_circ_0094342 is a diagnostic/prognostic biomarker for NSCLC and a therapeutic target for patients with NSCLC (Wang et al. 2021). Similarly, circHIF1A is upregulated in breast cancer (BC) tissues and plasma. When it regulates NFIB expression and transport through post-transcriptional and post-translational modifications, the AKT/STAT3 signaling pathway is activated and P21 is inhibited at the same time. In conclusion, circHIF1A may be a promising diagnostic marker for BC and a potential therapeutic target for BC therapy (Chen et al. 2021).

## Degradation of circRNAs

CircRNAs have better stability and longer half-life than linear mRNAs, but it doesn’t imply that circRNAs are not degradable. In fact, the ways of degradation differ due to the differences in their structure. For example, linear mRNAs have poly-A tail and m7G cap, and will be degraded by exonuclease when the two elements are removed (Łabno et al. 2016). Besides, mRNAs can also be degraded by endoribonuclease activities under some circumstances (such as NMD) (Lykke-Andersen and Jensen 2015; Popp and Maquat 2016; Silva and Romão 2009; Lindeboom et al. 2019). CircRNAs seem to hardly decay in theory as they have a covalently closed structure, but evidence demonstrates that circRNAs can be degraded in several ways (Fig. 2B).

### RNase L

RNase L is reported to decay circRNAs in several ways (Han et al. 2014; Liu et al. 2019b). To begin with, RNase L is encoded by the hereditary prostate cancer 1 (HPC1) locus and functions as an endoribonuclease. Second, it can be activated by 2-5A molecules of the formula [px5′A (2′p5′A)n; × 1 3; n > 2]. As 2-5A is produced from ATP when 2′-5′-oligoadenylate synthetase (OAS) is activated by double-stranded DNAs (dsDNAs), RNase L finally promotes the degradation of the invasive virus (Chakrabarti et al. 2011). It is reported that circRNAs are globally degraded by RNase L when cells are infected by the virus, which is essential for activation of PKR in the early stage of the innate immune response (Liu et al. 2019b).

Another research indicates that RNase L forms a crossed homodimer that is stabilized by ankyrin (ANK) and kinase homology (KH) domains. The homologous dimers located two kinases extended nuclease (KEN) regions for asymmetric RNA recognition, of which one works as KEN protomer that recognizes an identity nucleotide (U) while the other cuts RNA between nucleotides + 1 and + 2 (Han et al. 2014). Therefore, the synergistic effect of ANK, KH, and KEN domains causes RNase L-regulated and sequence-specific cleavage of virus and host RNA.

### Ago-dependent and Ago-independent degradation

Depending on whether Ago was necessary, circRNAs degradation can be divided into Ago-dependent and Ago-independent degradation. Evidence indicates that some miRs can cause the degradation of circRNAs (Kleaveland et al. 2018; Hansen et al. 2011). An example of this kind is miR-671, which is located in the nucleus and directs the Ago2-slicer-dependent cleavage of circCDR1 (Hansen et al. 2011).

It has been confirmed that drosophila GW182 and its human homologs (TNRC6A, TNRC6B, and TNRC6C) promote the degradation of circRNAs (Jia et al. 2019). When the three human homologs mentioned above were depleted, it was observed that accumulation of human circRNAs occurred, which is a solid proof of the function of GW182 as a promoter in circRNA degradation. Interestingly, GW182 protein contains an ago-binding domain (ABD), but GW182 mutants without ABD don’t affect circRNA degradation at all, which is different from the foresaid case. Later, further study suggests that it’s a middle region (Mid) in GW182 that functions as a molecular scaffold to recruit decay factors, and finally causes the degradation of circRNAs (Jia et al. 2019).

### Other possibilities

A recent discovery reveals that decay of RNA can be mediated under the structure-dependent mechanism depending on the overall base-pairing density of 3′UTRs (Fischer et al. 2020). mRNAs with highly structured 3′UTRs are globally degraded by RNA-binding proteins UPF1 and G3BP1while the degradation can be regulated by fusing the 3′UTR with an unstructured sequence to reduce the overall structure. Both proteins are critical in this progress. Similar to mRNAs, highly structured circRNAs were upregulated when both UPF1 and G3BP1 were downregulated (Fischer et al. 2020).

Interestingly, circRNAs can be detected in exosomes (Lasda and Parker 2016), which are formed by the segregation of intracellular poly vesicles with cell membranes and released to the extracellular space (Kalluri and LeBleu 2020). It is presumably a mechanism for circRNAs clearance that they are excreted into the extracellular space through extracellular vesicles (EVs) (Lasda and Parker 2016). Therefore, circRNAs can be degraded in the extracellular space through exosomes.

## Dynamic modification of circRNAs

Posttranscriptional modification has always been a center of attention. As a new type of non-coding RNA, circRNAs grant higher importance and value to the researches on them regarding post-transcriptional modification. Currently, such research is limited, but we managed to summarize the roles m6A modification plays in circRNAs, including regulating the biogenesis, nuclear export, translation and degradation of circRNAs. As illustrated in Fig. 3, m6A plays is significant to circRNAs.

### Promote the formation of circRNAs

As circRNAs are widely expressed in male germ cells and accumulated with the progress of sperm biogenesis, their existence offsets the degradation of mRNAs during spermatogenesis. Circular/linear ratios was observed to be upregulated in ALKBH5-null spermatogenic cells, while fewer circRNAs were identified in METTL3-null testes, suggesting that the level of circRNAs in male germ cells can be regulated by that of m6A (Tang et al. 2020). Further study indicates that reverse splicing tends to occur between m6A enrichment sites, usually near the start and end codons of linear mRNAs in spermatogenic cells (Tang et al. 2018; Tang et al. 2020). Additionally, pre-mRNAs with elevated m6A level appear to have tighter binding to the spliceosome, which reinforces back splicing (Tang et al. 2020; Xiao et al. 2016).

Another research reveals that METTL3 and YTHDC1 can regulate the biogenesis of circZNF609. When METTL3 and YTHDC1 are both or respectively depleted, it results in downregulation of circRNAs (Timoteo et al. 2020). Zhou et al. discovered that transposable elements (TEs) were more enriched in the flanking area of the circRNAs with m6A than those without m6A (Zhou et al. 2017). As it is revealed that METTL3/14 binds to TEs (Kelley et al. 2014) while the TEs in flanking introns of pre-mRNAs were validated to form a stem-loop in back splicing to facilitate the circularization (Zhou et al. 2017; Capel et al. 1993), it’s presumably the mechanism how m6A promotes biogenesis of circRNAs.

### Mediate the export of circRNAs

Researchers discovered that YTHDC1 could be combined with splicing factors and nuclear export adaptor protein SRSF3. Therefore, it is inferred that m6A mediates the export of methylated mRNA from the nucleus to the cytoplasm (Roundtree et al. 2017). Likewise, m6A also affects the export of circRNAs. In Chen’s research, it’s found that silencing YTHDC1 and METTL3 can both increase the expression of circNSUN2 in the nucleus. Moreover, the forced expression of YTHDC1 and METTL3 wild-type can save the nuclear exit defect of circNSUN2 caused by loss of YTHDC1 and METTL3. Therefore, nuclear export of circNSUN2 can be mediated by m6A modification (Chen et al. 2019a).

In addition, RNAs also affect nuclear export by their length. For example, long mRNAs (> 300 nt) are transferred by NXF1/NXT1 nuclear export receptor, a major export receptor for mRNAs. When intronless mRNAs are shortened to 130 nt or less, they will be exported through the U snRNA export pathway (Masuyama et al. 2004). Likewise, length-dependent export happens to circRNAs from nucleus to cytoplasm (Huang et al. 2018). Drosophila DExH/D-box helicase Hel25E is a necessary element in the nuclear export of circRNAs (> 800 nt) while the human homologs of Hel25E including UAP56 and URH49 also regulates the location of circRNAs in cells. UAP56 controls long circRNAs (> 1200 nt) and URH49 regulates short ones (< 400 nt). Therefore, the length of circRNAs determines the way of their nuclear export (Huang et al. 2018).

Furthermore, there is another way of export from the nucleus (Natalizio and Wente 2013). When the inner nuclear membrane comes into a bud which contains mature mRNAs, the bud subsequently fuses with vesicular of the outer nuclear membrane. During this process, mRNAs are exported to the cytoplasm (Natalizio and Wente 2013). Therefore, circRNAs might be exported from the nucleus by the bud, which, however, needs to be validated by further experiments. Notably, nuclear export can be affected by environmental factors such as heat shock (Natalizio and Wente 2013).

### Initiate translation of circRNAs

m6A is another driving element in cap-independent translation initiation. It’s common to find m6A circRNAs with coding potential in the human transcriptome (Zhou et al. 2017; Yang et al. 2017b). According to the finding that circRNAs usually contain large ORFs and start codons modified by m6A in the junctions (Tang et al. 2020), it can be implied that m6A would affect the translation of circRNAs in some way. In Yang’s research, only one m6A site was enough to drive translation initiation, while the production of protein was significantly reduced due to lack of m6A reader YTHDF3, which was subsequently discovered to have direct interaction with eIF4G2. Therefore, it’s YTHDF3 that recruits initiation factor eIF4G2 to activate translation (Yang et al. 2017b). During this process, it was demonstrated that translation is promoted by methyltransferase METTL3/14 and inhibited by FTO at the same time, indicating that translation driven by m6A can be regulated. To sum up, chances are that the junction m6A in the start codon serves as the IRES for translation initiation (Yang et al. 2017b).

### Mediate the degradation of circRNAs

Evidence indicates that m6A affects the degradation of some transcripts (Wang et al. 2014a, b; Du et al. 2016b). Du et al. discovered that the YTHDF2 N-terminal region could interact directly with the superfamily homology (SH) domain of CNOT1, subsequently decaying m6A RNA by recruiting the CCR4–NOT deadenylase complex (Du et al. 2016b).

It is reported that YTHDF2 mediates the degradation of circRNAs as well. Park et al. demonstrated that endoribonucleolytic cleavage could happen to both mRNAs and circRNAs containing m6A through YTHDF2-HRSP12-RNase P/MRP axis (Park et al. 2019). HRSP12, an adaptor protein, acts as a bridge to link YTHDF2 and RNase P/MRP (endoribonucleases). Hence, the complex composed of HRSP12, YTHDF2, and RNase P/MRP conducts subsequent RNA degradation. Their demonstration revealed that m6A circRNAs were preferential cellular substrates for RNase P/MRP-dependent degradation while HRSP12 was indispensable to bridge YTHDF2 and RNase P/MRP during such a degradation (Park et al. 2019).

### Others

Interestingly, circRNAs containing m6A are expressed in cell-type-specific patterns (Zhou et al. 2017). In Zhou’s study, m6A circRNAs in hESCs were different from those in HeLa cells. Both cells have their own characteristic circRNAs, circRASSF8 and circKANK1 for HeLa cells and circSEC11A and circTMEFF1 for hESCs, but all the four genes are expressed in both HeLa cells and hESCs. Thus, m6A circRNAs is hopefully a hallmark in cancers (Zhou et al. 2017). Notably, there is an article revealed that m6A modification can be regulated by circRNAs (Huang et al. 2020b). According to the article, overexpression of circSTAG1 lowered the import of ALKBH5 to the nucleus, which caused increase of m6A methylation. It’s believed that m6A functions in many aspects, but studies about m6A modification of circRNAs are limited. Therefore, it’s necessary to conduct more researches to profoundly reveal the effect of dynamic modification on circRNAs (Huang et al. 2020b).

## CircRNAs and cancers

As previously described in the review, circRNAs have various functions. Meanwhile, those functions make circRNAs important in the regulation of tumor progression. There has been extensive study about circRNAs in various cancers but their ways of regulation are far too complex and varied. Therefore, this review only summarized the mechanisms of circRNAs regarding their effect on tumor progression (Table1).

**Table 1:** Regulatory roles of circRNAs in cancers

Types of cancer	circRNAs	Roles of circRNAs in cancers	Expression
Hepatocellular carcinoma	cSMARCA5	Sponge miR-17-3p and miR-181b-5p	Low expression
	circFoxo3	Sponge miR-199a-5p	High expression
	hsa_circ_0051443	Sponge miR-331-3p via exosomes	Low expression
	circβ-catenin	Encode a novel protein β-catenin-370aa	High expression
Colorectal cancer	circERBIN	Sponge miR-125a-5p and miR-138-5p	High expression
	circPACRGL	Sponge miR-142-3p and miR-506-3p via exosomes	High expression
	circFNDC3B	Encode a novel protein circFNDC3B-218aa	Low expression
	circPPP1R12AA	Encode a novel protein circPPP1R12A-73aa	High expression
Gastric cancer	circMAPK1	Sponge miR-224	Low expression
	circAKT3	Sponge miR-198,	High expression
	circHuR	Sponge CNBP	Low expression
	circMRPS35	Serve as a scaffold to recuit KAT7 to Foxo1/3a promoter region	Low expression
	circSHKBP1	Sponge miR-582-3p via exosomes	High expression
Pancreatic cancer	circBFAR	Sponge miR-34b-5p	High expression
Gallbladder cancer	circERBB2	Regulate nucleolus localization of PA2G4	High expression
	circFOXP1	Sponge PTBP1 and miR-370	High expression
Breast cancer	circANKS1b	Sponge miR-148a-3p and miR-152-3p	High expression
	circFoxo3	Serve as a scaffold to bind to p53 and MDM2	Low expression
	circHER2	Encode a novel protein HER2-103 protein	High expression
	FECR1	Serve as an upstream regulator	High expression
Cervical cancer	circEYA1	Sponge miR-582-3p	Low expression
	circZFR	Sponge SSBP1	High expression
	circE7	Encode a novel protein E7	High expression
Ovarian cancer	circMUC16	Sponge miR-199a-5p and ATG13 protein	High expression
	circTNPO3	Sponge miR-1299	High expression
Prostate cancer	circFoxo3	Sponge miR-29a-3p	High expression
	circFoxo3	Bind to Foxo3 and inhibit EMT	Low expression
Lung cancer	FECR1	Sponge miR-584-3p	High expression
	circFoxo3	Sponge miR-155	Low expression
	circNDUFB2	Serve as a scaffold to bind to IGF2BPs and TRIM25	Low expression
	circSATB2	Sponge miR-326	High expression
Nasopharyngeal carcinoma	circCRIM1	Sponge miR-422a	High expression
	circTGFBR2	Sponge miR-107	Low expression
Bladder cancer	circPRMT5	Sponge miR-30c	High expression
	circRIP2	Sponge miR-1305	Low expression
	circFoxo3	Sponge miR-9-5p	Low expression
	circNR3C1	Sponge BRD4 protein	Low expression
Renal cell carcinoma	circPRRC2A	Sponge miR-514a-5p and miR-6776-5p	High expression
Glioblastoma	circASAP1	Sponge miR-502-5p	High expression
	circFoxo3	Sponge miR-138-5p and miR-432-5p	High expression
	circAKT3	Encode a novel protein AKT3-174aa	Low expression
	circFBXW7	Encode a novel protein FBXW7-185aa	Low expression
	circNFIX	Sponge miR-132 via exosomes	High expression

*CNBP* CCHC-type zinc finger nucleic acid-binding protein, *SSBP1* single-stranded DNA binding protein 1, *EMT* epithelial–mesenchymal transition, *BRD4* bromodomain-4 protein

### Tumors of the digestive system

#### Hepatocellular carcinoma

Hepatocellular carcinoma (HCC), a type of primary liver cancer, is now the second leading cause of cancer death worldwide (Wallace et al. 2015). CircRNAs have been reported to play a role in HCC regulation.

Firstly, circRNAs function as a sponge to regulate the progression of HCC (Yu et al. 2018; Huang et al. 2020c). Take circular RNA cSMARCA5 as an example. It is derived from exons 15 and 16 of the SMARCA5 gene, and inhibits HCC growth and metastasis by sponging miR-17-3p and miR-181b-5p to promote the expression of TIMP3, a well-known tumor suppressor (Yu et al. 2018). Opposite to the case above, circFoxo3 promotes the process of epithelial-mesenchymal transition (EMT) in HCC when it binds to miR-199a-5p and positively regulates ATP of binding to the subfamily C member 1 (ABCC1) (Huang et al. 2020c).

Secondly, circRNAs also play a key role in the progression of HCC through the exosomes (Huang et al. 2020d; Chen et al. 2020a). For example, hsa_circ_0051443, which is transmitted from normal cells to HCC ones via exosomes, regulates BAK1 expression by competitively binding to miR-331-3p, finally inhibiting malignant biological behavior by promoting apoptosis and blocking cell cycle (Chen et al. 2020a).

Thirdly, as mentioned in the review hereinabove, circRNAs act as translation templates to generate functional proteins. It has been revealed that circβ-catenin, derived from β-catenin, generates a novel isoform called β-catenin-370aa (Liang et al. 2019). When it physically interacts with GSK3β to phosphorylate β-catenin, a well-characterized oncogene in liver cancer, the degradation of β-catenin is triggered to promote the growth of liver cancer cells (Liang et al. 2019).

#### Colorectal cancer

Colorectal cancer (CRC), with a higher incidence in developed countries, is the third most common cancer and the fourth most common cause of cancer death in the world (Weitz et al. 2005). Fortunately, the roles circRNAs play in CRC have been identified.

Firstly, circRNAs regulate the progression of CRC by sponging miRs (Chen et al. 2020b; Xu et al. 2020b). For example, circERBIN promotes CRC proliferation, invasion, angiogenesis, and metastasis by targeting miR-125a-5p and miR-138-5p, which facilitates the expression of eIF4E-binding protein 1(4EBP-1). As a result, HIF-1α has its protein expression enhanced and pathway activated at the same time (Chen et al. 2020b).

Secondly, circRNAs play an important role in regulating CRC through the exosomes (Shang et al. 2020; Yang et al. 2020). For instance, the progression of CRC is facilitated when CRC-derived exosomal circPACRGL promotes the expression of the transforming growth factor-β1 (TGF-β1) by sponging miR-142-3p and miR-506-3p (Shang et al. 2020).

Thirdly, translation products from circRNA similarly influence CRC progression. For example, circFNDC3B-218aa, a novel protein encoded by circFNDC3B inhibits the progression by reducing Snail's inhibitory effect on FBP1 (Pan et al. 2020). Besides, circPPP1R12AA encodes a functional protein called circPPP1R12A-73aa then the product promotes the proliferation, migration, and invasion of colon cancer by activating the Hippo-YAP signaling pathway (Zheng et al. 2019).

#### Gastric cancer

Gastric cancer (GC), most usually found in Southeast Asian countries, features high mortality and low survival rates (Karimi et al. 2014). There are many studies on circRNAs in GC.

Firstly, circRNAs regulate the progression of GC via sponge function. Take for instance, circMAPK1 (hsa_circ_0004872) that binds to miR-224 and increases the expression of p21 and Smad4 (miR-224 target) by utilizing the function of sponge, resulting in inhibiting the proliferation, invasion and migration of GC cells (Ma et al. 2020). It is worth mentioning that circRNAs can affect the drug sensitivity of GC cells by sponging miRs (Huang et al. 2019a; Sun et al. 2020b; Peng et al. 2020). For example, circAKT3 that originates from exons 8, 9, 10, and 11 of the AKT3 gene is significantly up-regulated in Cisplatin (CDDP) resistant GC tissues and CDDP resistant cells. CircAKT3 modulates CDDP sensitivity by sponging miR-198 that inhibits PIK3R1 expression by activating the PI3K/AKT pathway in GC (Huang et al. 2019a). In addition to miRs, circRNAs also sponge proteins to affect GC. When circHuR interacts with CCHC-type zinc finger nucleic acid-binding protein (CNBP), it subsequently inhibits its binding to the HuR promoter, and at last down-regulates HuR and inhibits the progress of tumor (Yang et al. 2019).

Secondly, circRNAs act as scaffolds to regulate GC progression. CircMRPS35 is such an example that specifically binds to Foxo1/3a promoter region, then recruits KAT7 to the promoter region of Foxo1 and Foxo3a genes as a module scaffold to alter histone modification patterns, and subsequently increases H4K5 acetylation in its promoter region. It ultimately alters the expression of downstream genes p21, p27, Twist1 and E-cadherin, and inhibits the proliferation and invasion of GC cells (Jie et al. 2020).

Last but not least, circRNAs perform their regulatory functions by utilizing exosomes. For example, the exosome circSHKBP1 first regulates the miR-582-3p/HuR/VEGF pathway, and then inhibits HSP90 degradation and promotes GC progression in the end (Xie et al. 2020a).

#### Pancreatic cancer

As one of the leading causes of cancer death and one of the deadliest malignancies in the world, pancreatic cancer features inadequate response to most chemotherapy drugs and has a low 5-year survival rate (Vincent et al. 2011; Ilic and Ilic 2016).

Unfortunately, studies on pancreatic cancer are few and the existing ones suggest that circRNAs influence the progression of pancreatic cancer mainly by their sponging function (Guo et al. 2020; Kong et al. 2020a; Shi et al. 2020; Wong et al. 2020). For example, circBFAR upregulates the expression of the mesenchymal-epithelial conversion factor (MET) by sponging miR-34b-5p in cells. Meanwhile, it activates downstream AKT phosphorylation, which further activates the MET/PI3K/AKT signaling pathway and ultimately promotes the progression of pancreatic cancer (Guo et al. 2020).

#### Gallbladder cancer

Gallbladder cancer (GBC) is a malignant tumor of the biliary tract with poor prognosis and happens more often in developing countries (Sharma et al. 2017).

Additionally, it has been reported that circRNAs has an impact on GBC. For instance, when circERBB2 regulates nucleolus localization of PA2G4, it forms the circERBB2-PA2G4-TIFIA axis to upregulate Pol I activity and rDNA transcription in nucleolus, finally achieving promotion of GBC proliferation (Huang et al. 2019b). Besides, in another research, circFOXP1 promotes the Warburg effect when it upregulates the expression of PKLR by sponging PTBP1. It also sponges miR-370, the target of PKLR, to protect PKLR mRNA from degradation and ultimately promotes the progression of tumor (Wang et al. 2019a).

### Tumors of the genital system

#### Breast cancer

Breast cancer (BC) is a public health problem worldwide, and the death rate is still rising (Veronesi et al. 2005). In BC, circRNAs act as a sponge to regulate tumor progression (Zeng et al. 2018; Meng et al. 2020). For example, circANKS1b increases the expression of transcription factor USF1 when binding to miR-148a-3p and miR-152-3p. In turn, USF1 transcriptively upregulates TGF-β1 expression, and promotes EMT by activating TGF-β1/Smad signaling (Zeng et al. 2018).

In addition to sponge function, scaffold is another one that circRNAs utilize to affect BC progression (Du et al. 2017). Previous studies have shown that both p53 and Foxo3 are down-regulated by MDM2 in a proteasome-dependent manner. When circFoxo3 is upregulated during apoptosis of BC cells, it can bind to p53 and MDM2. Subsequently, it acts as a scaffold to further enhance the role of MDM2 in regulating p53 polyubiquitination while inhibiting MDM2 from regulating the polyubiquitination of Foxo3. This in turn increases the activity of Foxo3 and promotes the expression and cell apoptosis of its target gene Puma, thereby enhancing BC invasion (Du et al. 2017).

Besides, HER2-103, a protein encoded by circHER2 is demonstrated to have influence on BC progression (Li et al. 2020a). When it directly induces homo/hetero-dimerization of epidermal growth factor receptor (EGFR)/ HER3, the EGFR/HER3 signaling cascade is thereby reinforced, leading to AKT phosphorylation. Notably, triple negative breast cancer (TNBC) expressing HER2-103, is sensitive to Pertuzumab (Li et al. 2020a).

Another effect of circRNA on BC is to act as an upstream regulator to control its progression (Chen et al. 2018c). FECR1, a circRNA composed of FLI1 exon 4-2-3, binds to FLI1 promoter in cis and recruits TET1, a demethylase involved in DNA demethylation. It also binds to and down-regulates trans-methyltransferase DNMT1. As an upstream regulator to control BC progression, FECR1 drives the metastasis of BC by coordinating the regulation of DNA methylation and demethylase (Chen et al. 2018c).

#### Cervical cancer

Cervical cancer (CC) is the leading cause of death among women worldwide and occurs mainly due to persistent infection with the high-risk human papillomavirus (hrHPV) (Olusola et al. 2019).

When it comes to CC, circRNAs function as competitive endogenous RNA by sponging miRs/proteins. For example, by acting as a sponge for miR-582-3p, circEYA1 alleviates the inhibitory effect of CXCL14, and suppresses cell viability and colony formation while promoting cell apoptosis (Xu et al. 2020c). Besides, circZFR promotes the assembly of the CDK2/cyclin E1 complex when binding to SSBP1, which, in this process, acts as a scaffold protein to assemble and activate the CDK2/Cyclin E1 complex. The activated complex phosphorylates p-Rb and interrupts its pairing with E2F1. Subsequently, the released E2F1 transcription factor promotes the G1/S transition and the proliferation of CC cells after it triggers the transcription of genes associated with target DNA replication (Zhou et al. 2021).

Furthermore, it’s reported that circRNA functions as protein encoder to regulate CC progression. For example, circE7, originated from HPV and enriched in the cytoplasm, produces E7 oncoprotein in a cap-independent manner under heat shock conditions after m6A modification (Zhao et al. 2019).

#### Ovarian cancer

Ovarian cancer is the seventh most common cancer among women and the eighth most common cause of cancer death all over the world, with a five-year survival rate of less than 45% (Webb and Jordan 2017).

Its progression is affected by circRNAs mainly through the CE mechanism (Gan et al. 2020; Xia et al. 2020). For example, circMUC16 is highly expressed in ovarian cancer tissues and can bind to ATG13 protein. Besides, it also binds to miR-199a-5p and upregulates the expression of target Beclin1 and Runx1, which in turn promotes the transcription of circMUC16. Therefore, circMUC16 first promotes autophagy of ovarian cancer through Beclin1, Runx1 and ATG13, and then the progression of ovarian cancer (Gan et al. 2020). Besides, circTNPO3 acts as a sponge for miR-1299, of which the target gene is NEK2 (NIIMA associated kinase 2). The role circTNPO3 plays in the carcinogenesis of ovarian cancer is to promote PTX resistance in ovarian cancer cells by upregulating NEK2 expression via sponge miR-1299 (Xia et al. 2020).

#### Prostate cancer

Prostate cancer (PC) is the main non-skin cancer among men in many parts of the world. Its incidence and mortality vary between populations, but the former is high in developed regions (Rebbeck 2017).

There have been reports about circRNAs in PC due to their wide existence found in it through ultra-deep RNA-seq. Meanwhile, the degree of circRNA production is related to the progression of the disease (Chen et al. 2019b). CircFoxo3 is high expressed in PC tissues and serum. It promotes the progression of PC after up-regulating the expression of solute vector family 25 member 15(SLC25A15) by acting as a sponge for miR-29a-3p (Kong et al. 2020b). Conversely, it’s been reported at the same time that circFoxo3 can inhibit the progression of prostate cancer (Shen et al. 2020). According to Shen’s research, circFoxo3 is low expressed in high-grade PC. Studies have shown that low expression of circFoxo3 promotes PC progression and chemical resistance to docetaxel by enhancing EMT, suggesting that circFoxo3 inhibits PC progression (Shen et al. 2020). The reason for the contrary results probably lies in the difference in sampling criteria or insufficiency of samples, or both (Zhang et al. 2021).

### Tumors of the respiratory system

#### Lung cancer

The incidence of lung cancer is on the rise internationally. Smoking is the biggest risk factor for such a disease while non-smokers will have their risk of lung cancer increased by 20% at least thanks to constant exposure to environmental tobacco smoke, A.K.A. secondhand smoking (Bade and Dela 2020; Schwartz and Cote 2016).

In lung cancer, circRNAs function as a sponge to control tumor progression (Li et al. 2019; Zhang et al. 2018b; Cheng et al. 2019). For example, FECR1 promotes tumor metastasis by activating the ROCK1 pathway via adsorption of miR584-3p (Li et al. 2019). As circFoxo3 is down-regulated in NSCLC and activates Foxo3 gene by binding to miR-155, it finally inhibits the proliferation and invasion of NSCLC cells (Zhang et al. 2018b).

CircRNAs can additionally act as a scaffold to regulate lung cancer progression (Li et al. 2021a; Liang et al. 2021). For example, circNDUFB2, acting as a scaffold, binds to IGF2BPs and TRIM25 first and then forms a ternary complex. In this complex, the interaction between TRIM25 and IGF2BPs is strengthened, which thereby promotes the ubiquitination degradation of IGF2BPs (Li et al. 2021a). Moreover, circNDUFB2 also inhibits tumor growth after it recruits immune cells into TME by activating the RIG-I-MAVS pathway (Li et al. 2021a).

CircRNAs can affect lung cancer via exosomes in the meantime (Zhang et al. 2020b). When circSATB2 participates in intercellular communication via exosomes, it promotes the proliferation, migration and invasion of NSCLC cells as well as inducing the abnormal proliferation of normal bronchial epithelial cells (Zhang et al. 2020b).

Notably, it’s been demonstrated that some circRNAs originating from fusion genes also facilitate the progression of lung cancer (Wu et al. 2019b; Tan et al. 2018).

#### Nasopharyngeal carcinoma

The incidence of nasopharyngeal carcinoma (NPC) is geographically distributed, mainly prevalent in East and Southeast Asia, with decreasing morbidity and mortality though (Chen et al. 2019c).

CircRNA mainly acts as a sponge to regulate NPC progression (Hong et al. 2020; Li et al. 2021b). For example, circCRIM1 competitively binds to miR-422a and prevents miR-422a from inhibiting its target gene FOXQ1. As a result, it causes NPC metastasis, EMT, and docetaxel chemical resistance (Hong et al. 2020). As circTGFBR2 inhibits miR-107, TGFBR2 expression is up-regulated and the NPC process is inhibited (Li et al. 2021b).

### Tumors of urinary system

#### Bladder cancer

Bladder cancer is one of the most common urinary malignancies. It is a heterogeneous disease with a variable natural history and high mortality and morbidity (Kirkali et al. 2005; Torre et al. 2012).

Its tumor progression can be accelerated by circRNAs when the latter sponges miRs. For example, upregulation of circPRMT5 acts as a sponge for miR-30c and modulates the Snail1/E-cadherin pathway to promote EMT in bladder cancer cells by competing with miR-30c (Chen et al. 2018b). Sponging miRs enables circRNAs to inhibit bladder cancer progression additionally. CircRIP2, low expressed in bladder cancer, has been demonstrated to have effects on suppression of tumor progression when circRIP2 binds to miR-1305 to enhance TGF-β2 and induces EMT via the TGF-β2/Smad3 pathway (Su et al. 2020). Likewise, circFoxo3 is downregulated in bladder cancer tissues and promotes TGFBR2 expression by binding to miR-9-5p, which inhibits the growth and metastasis of bladder cancer cells in the end (Li et al. 2020b).

Furthermore, circRNAs can regulate bladder cancer progression when they sponge proteins. Studies have shown that c-Myc is enabled to act as a transcription factor for the progression of bladder cancer after BRD4 forms a complex with the c-Myc promoter (Wu et al. 2016). As an endogenous blocker, circNR3C1 binds to the BRD4 protein first, and then disconnects the formation of the BRD4/c-Myc complex, ending up inhibiting the progression of bladder cancer (Xie et al. 2020b).

#### Renal cell carcinoma

Renal cell carcinoma (RCC) is the third leading cause of death among urological tumors (Jemal et al. 2008), and its incidence increases with age (Ljungberg et al. 2015).

RCC is regulated by circRNAs mainly through the function of the latter as sponge. For example, circPRRC2A sponges miR-514a-5p and miR-6776-5p in the beginning, subsequently prevents the degradation of the tissue-specific oncogene TRPM3, and finally promotes the progression of RCC (Li et al. 2020c).

### Tumors of the nervous system

#### Glioblastoma

Glioblastoma (GBM) is the most common malignant tumor of the central nervous system. The incidence of GBM increases with age but the prognosis is poor. The 5-year survival rate of GBM is less than 10% (Dolecek et al. 2012).

Similar to the cases in other cancers, circRNAs function as a sponge to regulate GBM. For instance, circASAP1 is highly expressed in GBM. When it activates NRAS/mitogen-activated protein kinase kinase 1(MEK1)/extracellular signal-regulated kinase 1 and 2 (ERK1–2) signals by sponging miR-502-5p, GBM progression and temozolomide resistance is ultimately facilitated (Wei et al. 2021). As a ceRNA, circFoxo3 binds miR-138-5p and miR-432-5p in the first place, then increases the expression of target gene activated T cell 5 (NFAT5), and finally activates the oncogenic activity of circFoxo3 in promoting the proliferation and invasion of GBM cells (Zhang et al. 2019). Likewise, when circFoxo3 is upregulated during oxidative damage in neurodegenerative diseases, it causes Foxo3 to be transposed to nucleus and promotes mitochondrial apoptosis of HT22 cells. Therefore, mitochondrial apoptosis pathway will be inhibited and HT22 cells protected from oxidative damage if circFoxo3 is silenced (Lin et al. 2020).

It’s worthy of note that translation of circRNAs in GBM seems more common than in other cancers, and that products of translation always have effect on regulation of GBM (Kong et al. 2020c). For example, AKT3-174aa, a novel protein encoded by circAKT3, competitively interacts with phosphorylated PDK1 when acting as a molecular decoy. By doing so, it reduces AKT-Thr308 phosphorylation, and finally inhibits GBM cells in the aspect of proliferation, radiation resistance, and tumorigenicity in vivo (Xia et al. 2019). Likewise, FBXW7-185aa, encoded by circFBXW7, decreases the half-life of c-Myc as it antagonizes the stabilization of c-Myc, which is induced by USP28. When FBXW7-185aa is up-regulated, it inhibits GBM cells with respect to proliferation and cell cycle acceleration. Oppositely, it promotes GBM in both vitro and vivo with respect to malignant phenotype when it is down-regulated (Yang et al. 2018).

Exosomes are the other way for circRNAs to regulate GBM. For example, circNFIX is an exosome from temozolomide (TMZ) drug-resistant cells. It increases TMZ resistance in GBM via miR-132 sponge, which accelerates the progression of GBM at last (Ding et al. 2020).

## Detecting methods of circRNAs

As we discussed above, circRNAs have great prospects in clinic as potential tumor biomarkers and therapeutic targets. However, due to the lack of sensitive and specific detecting methods, the clinical application of circRNAs is obviously limited. Conventional RNA analysis methods, such as Northern Blot and gel electrophoresis, are of low sensitivity and thus not suitable for the determination of circRNAs. At present, reverse transcription polymerase chain reaction (RT-PCR), a sensitive and specific detecting method, is widely applied in the study of circRNAs. However, results of RT-PCR may be false positive because of the strand displacement of reverse transcriptase (RT) (Kelleher and Champoux 1998) and import of template switching artifacts (Luo and Taylor 1990; Cocquet et al. 2006). In fact, there are some other specific methods to detect circRNAs, which are summarized as follows.

### Oligonucleotide

An oligonucleotide molecular (oDNA) probe that is complementary to the target circRNA sequence is immobilized on the chip to detect target circRNA. For example, an oDNA probe, complementary to the circNFIX sequence, was fixed on the SOI-NR chip. Then the biosensor detected the level of circNFIX, a potential biomarker of glioma, with a range of 10^–17^ M to 10^–15^ M (Ivanov et al. 2021a). Similarly, h–k-SOI-NW chip fixed with oDNA probe that was complementary to glioma associated circSHKBP1 sequence, and real-time detection of circSHKBP1 was ~ 10^–16^ M (Ivanov et al. 2021b). Both above have high sensitivity and short detection time (within 15 min and 400 s (~ 7 min), respectively).

### Rolling circle amplification

Rolling circle amplification (RCA) uses DNA as a template to form single-stranded DNA or RNA under the action of DNA or RNA polymerase. The rolling-circle transcription catalyzed by RT greatly improves the sensitivity of RCA for the detection of circRNAs. The method based on these two theories is named reverse transcription-rolling circle amplification (RT-RCA) (Boss and Arenz 2020; Liu et al. 2020). RT-RCA is specific and sensitive. It can distinguish circRNAs from corresponding linear transcripts and detect target circRNA as low as 1.1 fM (Liu et al. 2020). On top of that, RT-RCA can also be combined with thermostatic netlike hybridization chain reaction (HCR) to detect genes containing repeated sequences, such as telomere DNA, centromeric DNA and ribosomal DNA (rDNA) (Dong et al. 2021).

### Specific probes or primers

Specific probes or primers, designed based on junction sites of circRNAs, can also be applied for determination of circRNAs. In Zhang’s research, two designed DNA probes were accurately connected by splint ligase at the junction site of circRNA and then the DNA probes were amplified by PCR (Zhang et al. 2020c). The detecting method can directly distinguish circRNAs from corresponding linear transcripts and the lower limit of detection is 1 fM. Since only one circRNA can be detected at a time, it seems not conducive to high-throughput screening. Likewise, a SLP induced double exponential amplification method was established by designing a pair of stem-loop primers (SLP) to accurately identify circRNA junction sequences. Only in the presence of circRNAs can two SLPs successfully form a double stem loop structure DNA and trigger subsequent exponential amplification (Zhang et al. 2020d). The method can also distinguish between circRNAs and linear RNAs directly and detect target circRNAs as low as 10 aM. Moreover, only 1 ng total RNA sample will be enough when detecting specific circRNAs.

### Other methods

In addition, using “miR sponge” properties of circRNAs and the advantages of duplex-specific nuclease (DSN), a dual signal amplification strategy is created for rapid and sensitive detection of circRNAs (Jiao et al. 2018). All that is needed is to mix molecular beacon probes, circRNAs and DSN enzyme and incubate for 30 min. The detection limit is about 10 fM. This method is simple neither complex equipment and procedures nor external modification of circRNAs is needed.

### Future perspective

Nowadays, the translatable potential of circRNAs greatly catches the eyes of researchers, raising a challenge for the conventional definition of ncRNAs. It’s confirmed that circRNAs are translated in a cap-independent way, during which IRES or other initial elements perform the same functions as eukaryotic initiation factors in a cap-dependent way. Nevertheless, the efficiency of cap-independent translation is merely 1–10%, much lower than that of cap-dependent translation (Merrick 2004; Legnini et al. 2017). It’s found that cap-independent translation is significant and indispensable under the condition of various stresses such as heat shock and at the same time has the potential to be a guarantee for essential life activity (Zhou et al. 2015; Yang et al. 2017b; Legnini et al. 2017). It still remains unknown whether cap-dependent and cap-independent initiation works simultaneously or alternatively. Therefore, it’s of great importance to explore this complication in future research.

As summarized above, there are several ways to degrade circRNAs, but more yet to be discovered. Since circRNAs are stable in vivo and sometimes carcinogenic, it was considered in this paper whether it would be possible to find a way to decay targeted carcinogenic circRNAs. Once the possibility is confirmed, and this would be a potential treatment in clinic.

m6A modification is widespread in circRNAs while m6A circRNAs have been discovered in various cells (Tang et al. 2020; Zhou et al. 2017). m6A modifications are written and read by protein complexes interacting with mRNA, but many m6A modification sites in circRNAs differ from those in mRNAs (Zhou et al. 2017). In addition, it is reported that m6A circRNAs can affect the immune reaction. Chen et al*.* demonstrated that the circRNAs without m6A modification induced antigen-specific T and B cell responses, but the ones modified by m6A could prevent it because they were marked by m6A as self circRNAs (Chen et al. 2019d).

Currently, most tumor biomarkers used in clinic are proteins with low organ specificity. For example, CEA is common in gastrointestinal tumors and CA19-9 in various adenocarcinoma. CircRNAs are stable and specific, and can identify and locate the tumor rapidly and accurately when combing with other biomarkers or other examination methods, such as computerized tomography (CT), B ultrasound, gastroscopy, colonoscopy, etc. Although many studies have demonstrated the potential of circRNAs as tumor biomarkers and therapeutic targets, it is not time for circRNAs to be applied in clinic yet given the following reasons. Firstly, most of the existing studies exploring the potential of circRNAs as tumor biomarkers are limited by their sample size, and the diagnostic sensitivity and specificity need to be further verified. Secondly, many aspects of circRNAs such as naming system, sample processing, detection method have not been standardized, which is not convenient to analyze existing results unifiedly and adds to the difficulty of their clinical application as tumor biomarkers (Wen et al. 2020). Thirdly, the concentration of circRNAs in body fluids is unknown and poorly studied, and the reference interval for circRNAs in specific diseases has not been determined. What's more, specific methods for the determination of circRNAs that are suitable for widespread clinical application are still under study. Fourthly, numerous studies have revealed the regulatory roles of circRNAs as therapeutic targets in tumors. However, it is a challenge to deliver circRNAs effectively to target lesions. Problems like off-target effects, the stability and immunogenicity of transport medium (such as exosomes and nanomaterials), and other unknown side effects must be taken into account (He et al. 2021b). Future studies should be conducted with larger sample size under the same standard to assess the potential of circRNAs as a biomarker. Moreover, except for circRNAs, there are other circulating RNAs in body fluids such as miRNAs, lncRNAs and mRNAs. In addition, there are many overlapping sequences between circRNAs and corresponding linear transcripts. Therefore, it is necessary to develop specific methods for the detection of circRNAs. Besides, although several studies have revealed the concentration of circulating RNA in the blood or in other biological materials in a particular disease (Additional file 1: Table S1), there are few reports about circRNAs and future studies could focus on this way. Finally, the regulatory roles of circRNAs in tumors have been revealed gradually, and future researches should focus on how to utilize their therapeutic targets roles for treatment. Overall, there is still a long way for circRNAs to be applied in clinical practice.

## Conclusions

CircRNAs are a novel kind of ncRNAs resulted from back splicing of pre-mRNAs. Compared to mRNAs, circRNAs are more stable due to their circular structure, which in turn enables them to serve as biomarkers. In the meantime, there are ways to make them degraded, including RNase L, Ago-dependent, and Ago-independent degradation. According to existing reports, m6A is significant to circRNAs with respect to formation promotion and translation initiation as well as the mediation of nuclear export and degradation. Moreover, circRNAs have significant roles to play in the regulation of tumor progression thanks to their various functions. Currently, methods that can specifically detect circRNAs have been reported gradually.

In conclusion, this paper presents a fundamental overview of circRNAs regarding biogenesis, biomarkers, functions, degradation, and dynamic modification, aiming to provide a general and profound understanding of circRNAs. Moreover, it summarizes the roles of circRNAs in various cancers and the specific methods to detect circRNAs.

## Supplementary Information

**Additional file 1: Table S1.** Detecting methods of circulating RNAs.

## Data Availability

Not applicable.

## Abbreviations

CircRNAs: Circular RNAs
eIF: Eukaryotic initiation factor
HHRs: Hammerhead ribozymes
IRESs: Internal ribosome entry sites
m6A: N-6 methylation
ORF: Open reading frame
ncRNAs: Non-coding RNAs
LTRs: Long terminal repeats
mRNA: Message RNA
circCDR1: Cerebellar degeneration-related protein 1
RBP: RNA binding protein
MBL/ MBNL1: Muscleblind
miR: MicroRNA
TNRC6A/B/C: Trinucleotide repeat-containing 6A/B/C
QKI: Quaking
RCA: Rolling circle amplification
EMT: Epithelial-mesenchymal transition
RT: Reverse transcriptase
HCC: Hepatocellular carcinoma
CRC: Colorectal cancer
GC: Gastric cancer
GBC: Gallbladder cancer
BC: Breast cancer
CC: Cervical cancer
PC: Prostate cancer
NPC: Nasopharyngeal carcinoma
RCC: Renal cell carcinoma
GBM: Glioblastoma
