# Supplementary material for: CircRNAs and their regulatory roles in cancers
Source: Mol Med. 2021 Aug 26;27:94. doi: 10.1186/s10020-021-00359-3 (PMC8393742; doi:10.1186/s10020-021-00359-3)
Supplement: Supplementary file 1 — Additional file 1: Table S1. Detecting methods of circulating RNAs. [file 10020_2021_359_MOESM1_ESM.docx]

| **Additional table 1. Detecting methods of circulating RNAs** | | | | | |
| --- | --- | --- | --- | --- | --- |
| Diseases | Objects | Concentration | Samples | Detecting methods | PMID |
| Healthy persons | RNA | 144 ± 22 mg/l | plasma | Fluorometric method | 5026765 |
| Early-onset preeclampsia | RNA | 0.1~5 ng/ml | plasma | Transcriptome enrichment | 32611681 |
| Breast cancer | RNA | 1.2 ± 0.9 ng/ml | plasma | SYBR Green II assay | 17108228 |
| Pregnancy-related hypertension | miRNA | CHT: 0.22~0.38 ng/µl; GHT 0.16~0.91 ng/µl; mPE: 0.42~1.85 ng/µl; sPE: 0.25~8.21 ng/µl. | plasma exosomes | Qubit 2.0 fluorometer & miRNA specific fluorescence analysis | 29153681 |
| Lung cancer | hnRNP B1 mRNA | 0.99 ± 0.26 pg/μg plasma RNA | plasma | RT-PCR | 15777973 |
| Renal cell carcinoma | RNA | conventional RCC: 1414.19 ± 91.95 ng/ml; renal oncocytomas: 560.71 ± 69.54 ng/ml. | serum | Spectrophotometric method | 18383864 |
| Angina pectoris | vaspin mRNA | SAP: 1.19 ± 0.85; UAP: 0.82 ± 0.56. | PBMC | SYBR Premix Ex TaqTM | 21913793 |

Note: CHT, chronic hypertension; GHT, gestational hypertension; sPE, severe preeclampsia; mPE, moderate preeclampsia; RCC, renal cell carcinoma; SAP, stable angina pectoris; UAP, unstable angina pectoris; PBMC, peripheral blood mononuclear cell.
